# How to Implement: The Need to Address a Fundamental Weakness in the Science of Integrated Care

**DOI:** 10.5334/ijic.9852

**Published:** 2025-06-27

**Authors:** Nick Goodwin

**Affiliations:** 1 University of Newcastle and Honorary Conjoint Scholar, Central Coast Local Health District, Gosford, NSW, Australia

**Keywords:** evaluation, impact, implementation, integrated care

In 1999, at the time of the birth of the International Journal of Integrated Care, Walter Leutz wrote a seminal article in *The Milbank Quarterly* that formulated Five Laws for integrating medical and social services (**Box 1**) [1]. Moreover, Leutz offered three recommendations for action: to involve the users and providers early in the planning process; to develop systems to integrate services; and to clarify the borders between medical and other systems. Research on integrated care models over the past 25 years has generally supported these Laws [2].

**Box 1 The Original Five Laws for Integrated Care [1]**

Law 1: You can integrate all of the services for some of the people, some of the services for all of the people, but you can’t integrate all of the services for all of the people
Law 2: Integration costs before it pays
Law 3: Your integration is my fragmentation
Law 4: You can’t integrate a square peg into a round hole
Law 5: The one who integrates calls the tune

However, our understanding has also advanced, for example: from ‘involving’ users to the need for people-centred care and co-productive partnerships with them; from developing mechanisms to link services, to the more fundamental curation of multi-disciplinary teams and complex adaptive systems that begin to make previous professional boundaries obsolete; and from clarifying borders between services, to breaking these borders down to reduce the ever increasing numbers of people living with complex needs from falling through the gaps in care.

Having now reached its 25^th^ year, IJIC has contributed much to the advancement of the science of integrated care globally. Yet, as our 10^th^ and 20^th^ anniversary editorials observed, adopters still ‘don’t know how’ best to implement integrated care in both policy and practice, exacerbated by a lack of measurable outcomes and an understanding of its impact [3,4]. This represents a fundamental weakness of the integrated care movement. Certainly, integrated care continues to advance and flourish in many regions and countries, but it has taken a backward step in others.

My adopted home in New South Wales, Australia, is a case in point. A strategic commitment to promote integrated care by NSW Health has been in place since 2014 [5], including a strategic framework published in 2018 [6]. Major programs, such as the Integrated Care Demonstrators [7] and Collaborative Commissioning [8] were created to support the strategy, including leadership and professional support programs curated from key institutional pillars for quality improvement such as the NSW Agency for Clinical Innovation.

Yet, over the last year, political commitment at State level towards integrated care as policy has imploded, most of the policy mechanisms to promote it weeded out, and ‘integrated care’ as a term become very much out of favour. This experience represents a fundamental lack of current belief at policy-level in the promise of integrated care linked to an inability to demonstrate its impact and value. Yet it also represents what is likely to happen when Leutz’s fourth Law _ that you cannot integrate a square peg into a round hole _ is ignored.

In describing this Law, Leutz primarily explained the problem through funding mechanisms in which health care organisations (largely responsible for acute care – the square peg) attempted to develop policies and mechanisms to influence long-term care and other sectors (largely responsible for social care – the round hole). In essence, Leutz observed that policy making and management to the design, oversight and administration of integration initiatives was most likely to fail when driven from a singular entity as opposed to a collective.

Much has been written and demonstrated to show that institutionally led approaches that do not effectively embrace co-productive partnerships with other sectors and with the community are likely to be less successful [9,10]. The peculiarities of institutional politics, governance and regulatory rules, financial imperatives, embedded professional and managerial norms and values, all create immovable boundaries that make any foray into integrated care problematic. Expecting one paradigm (usually the health and medical one) to drive forward integrated care across multiple others will likely set ablaze the mismatch of motivations between partners rather than curate a common path. Yet in the aftermath of a short-lived program, the architects of the square peg are more likely than not to claim that the round hole was the problem.

Our 20^th^ anniversary editorial concluded that this prevailing ‘top-down’ approach to implementation lies at the heart of the many failures of integrated care programs [4]. The square peg – round hole syndrome is also a failure to learn from history driven by an inability to acknowledge and be open about when things do not work and the lack of commitment to long-term sustainable change [11]. Yet, it must be said, the current science behind integrated care remains weak in providing the essential guidance required by policymakers and system designers to make more effective choices.

This editorial began by saying that policymakers still ‘don’t know how’ to best implement integrated care, but that is not exactly true. There are more than enough frameworks to help understand what the essential building blocks of integrated care should comprise and the issues to be addressed to lead and manage them. But within such knowledge lies an essential gap between recognising the nature and complexity of what needs to be addressed, to the more detailed and granular guidance on how to get things done [12].

To put it another way, the science has mapped out the ocean on which integrated care must sail and provided knowledge on the nature of the ship and its crew that will set out on its journey. What is hasn’t provided is enough practical guidance and knowledge on how to navigate the seas ahead. Implementation science tells us that leadership will matter, that networks and relationships need to be curated and sustained, that cultural differences and variations in norms and values require attention, that the journey will require time to complete, that it will require team work and co-productive practices, that flexibility and adaptation to an ever-changing environment over time will be needed, that our maturity to navigate the seas ahead will come with practice and effort. All of this is likely to be true, but it is abstract and provides little that is tangible.

In celebrating the Journal’s 25^th^ anniversary, and for all the advances and collective knowledge that has been developed along the way, a core challenge ahead is to address the weakness the integrated care movement has in understanding ‘how to’ implement [13]. Moreover, given that the success and failure of integrated care programs is as much in ‘how’ you do things, as ‘what’ you do, future evaluations must factor in these implementation components when assessing impact. Without this knowledge, evaluations that provide evidence for the value of integrated care programs cannot be easily replicated or their results misinterpreted. By extension, it then becomes too easy for past implementation mistakes to be repeated.

Governments internationally will continue to drive forward integrated care policies and programs. In Australia, as NSW Health has seemingly taken a step back, the Australian Government has recently announced a new Integrated Care and Commissioning Initiative to trial new models of care that bring health, aged care, disability and veteran’s care resources to support rural, remote and First Nations communities [14]. The intention is to build community-led and local solutions in its pilot sites across Australia. Overcoming the square peg – round hole syndrome will much depend on how these programs are designed and driven in ways that are compatible with their specific environments.

As a final thought, the role of external organisations to work alongside systems to effectively design and adopt integrated care programs, such as through the International Foundation for Integrated Care [15], is likely to be important since no major policy program can be executed effectively without such knowledge, mentorship and guidance. The role of our Journal must be to continue to provide the scientific bedrock on which such advice is based, and that means our next phase must seek to address our weaknesses and enhance our collective understanding of both implementation and impact.

## Editor Note

After 25 years’ service, Professor Nick Goodwin has stepped down as co-Editor in Chief of the International Journal of Integrated Care. Nick will continue to serve as Editor and will lead the upcoming special issue on Understanding the Impact of Integrated Care.
